# Mechanisms influencing spatiotemporal differentiation of tourist towns based on geographic detector: A case study of Fujian Province

**DOI:** 10.1371/journal.pone.0298078

**Published:** 2024-04-04

**Authors:** Xiuzhi Lin, Qiuqin Zheng, Kai Su, Qiuhua Chen

**Affiliations:** 1 College of Tourism and Leisure Management, Fujian Business University, Fuzhou, Fujian, China; 2 Anxi College of Tea Science, Fujian Agriculture and Forestry University, Fuzhou, Fujian, China; 3 College of Economics and Management, Fujian Agriculture and Forestry University, Fuzhou, Fujian, China; Qufu Normal University, CHINA

## Abstract

The construction of tourist towns is an important aspect of new-type urbanization construction. In this study, 155 tourist towns in Fujian Province were selected as samples to analyze spatiotemporal differentiation using the geographical concentration index, nearest neighbor index, and local correlation index. Then, a geographic detector model was used to detect the factors that influence the spatiotemporal differentiation of tourist towns and to analyze the explanatory power and interaction of these detection factors. Finally, the mechanisms underlying the detection factors were discussed. Factors affecting the spatiotemporal differentiation of tourist towns in Fujian Province were core factors of traffic network, level of urbanization and population distribution; important factors of industrial structure and socioeconomic basis; and a fundamental factor of policy guidance. These six factors interacted to jointly affect the spatiotemporal differentiation of tourist towns in Fujian Province. The results of this study can provide a basis for the development of tourist towns in other similar regions and have reference value for better optimizing the pattern of urban and town systems and coordinating the synergistic development of urban and rural areas.

## 1. Introduction

The integration of urbanization construction and tourism promotes the agglomeration of the regional population, resources, and funds and leads to the formation of the characteristic tourist towns [1]. Tourist towns combines tourism development with small-town construction, and the tourism industry occupies a pillar or dominant position in the local industrial system. Such towns can promote the integrated development of various industries in a region, create socioeconomic benefits, promote the construction of an ecological environment, and assist in cultural inheritance and protection [2–4]. Moreover, tourist towns with reasonable spatial layouts are conducive to forming an industrial agglomeration effect, promoting coordinated regional development among regions and urban construction.

Due of their significant economic, environmental, and social benefits, tourist towns have developed rapidly since the 1870s. Britain, the United States, Japan, Germany, and other countries have developed tourist towns [5–9]. In China, Yunnan Province took the lead in proposing the strategic concept of a "tourist town" in July 2005. In May 2006, the Ministry of Construction and National Tourism Administration jointly held the "National Conference on the Development of Tourist Towns" in Yunnan Province. As a result, the research and construction of tourist towns have been formally incorporated at the national level [10].

The National Development and Reform Commission of China also supports small towns with geographical advantages or unique resources to develop into professional towns with advanced manufacturing, transportation hubs, commercial circulation, cultural tourism, and other functions. The construction of tourist towns, as an important component and approach to new-type urbanization construction, has been widely valued by governments at all levels of the worldwide. However, the construction of tourist towns is still in the initial stage, with unbalanced development of regional spatial structures and poor connectivity, which is not conducive to sustainable development [11]. Moreover, the construction of small towns has many problems, such as a large number, small scale, weak service functions, and insignificant regional agglomeration effects, which also pose severe challenges to the construction of tourist towns [11]. To achieve breakthroughs in the construction of tourist towns within the existing urban construction framework, a reasonable spatial structure must be constructed. Therefore, it is of great practical significance to evaluate the factors that influence the spatiotemporal differentiation of tourist towns and explore the mechanisms that influence constructing a reasonable spatial layout of tourist towns, thereby promoting the sustainable development of the regional tourism industry, and the construction of high-quality regional urbanization.

This study focuses on the following problems:(1) exploring the characteristics of the spatiotemporal differentiation of tourist towns in Fujian Province; (2) exploring the degree of influence of each influencing factor on the spatiotemporal differentiation of tourist towns in Fujian Province; (3) exploring the mechanisms of influence regarding the spatiotemporal differentiation of tourist towns in Fujian Province. Thus, this study can provide a reference for decision-makers to better optimize the pattern of urban and town systems and can also provide experience for the development of tourist towns in other similar regions.

## 2. Literature review

With the development of tourism and the acceleration of urbanization, academic research on tourist towns has become increasingly in-depth and has achieved more systematic research findings. In terms of content, studies have primarily focused on planning and management [12,13], evaluation [14,15], community involvement [16–18], ecological environment [19–22], the coordinated development of urban construction and tourism [1,23], and sex secrecy and risk [24,25]. The few studies of the spatial distribution of tourist towns have primarily explored the characteristics of spatial distribution, influencing factors, and influencing mechanisms.

Researchers have used different GIS spatial analysis methods to study the spatial distribution characteristics of tourist towns. Zhan et al [26] analyzed the spatial distribution characteristics of the 100 most representative rural tourist towns in China using kernel density estimation, an imbalance index, and the Gini coefficient. Sun and Wang [27] studied the overall distribution characteristics of characteristic landscape tourist towns and villages through the nearest-neighbor distance hierarchical analysis, kernel density analysis, and inverse distance weighted spatial interpolation. Yang et al [28] used a participatory rural appraisal methodology, GIS spatial analysis, and high-definition remote-sensing imagery to explore the spatiotemporal characteristics of the functional transformation and restructuring of tourist towns in the Huangshan Mountains. Xu et al [29] adopted the radius of gyration, spatial distribution curve, and discrete index methods to reveal the spatial structural characteristics of tourist towns in Shanghai. Tang and Ma [30] explored the spatial structural characteristics of a Huxiang-style cultural tourist town using the closest neighbor index, kernel density estimation, imbalance index, and hotspot analysis.

Research on the factors that influence the spatial distribution of tourist towns has primarily combined qualitative and statistical analyses. Zhan et al [26] noted that national development strategy, social environment, geographic environment, and history influence the spatial distribution of rural tourist towns. Sun and Wang[27] found that the distribution of characteristic landscape tourist towns and villages is closely related to markets, scenic spots, transportation, and population. Iordache and Cebuc [31] noted that town planning influences the formation of spatial patterns of tourist towns. Lv et al [32] noted that transportation accessibility influences the spatial distribution of ancient tourist towns. Tang and Ma [33] indicated that the economic base, population density, urbanization level, high-level scenic spots, market demand, and consumption characteristics are the main driving factors that drive the spatial distribution of Hunan-style cultural tourist towns. Ma and Li [34] noted that the factors that affect the spatial distribution of characteristic tourist towns are the natural environment, tourism economy, location, and transportation.

Researchers also explored the influence mechanism of the spatial distribution of tourist towns. Xiang et al [35] used Sangu tourist towns in Wuyi Mountain as an example and analyzed the driving mechanisms that have driven the evolution of its spatial structure in terms of both the extrinsic drive of the factors of the tourism industry, tourism market, policy, planning, science and technology, and tourist behaviors, and intrinsic adaptations to the natural topography, land resources, and transportation location. Xi et al [36] noted regarding land-space utilization in growing tourist towns, the natural geographic environment frames the basic direction, the sustained growth of tourism demand is the fundamental driving force, the rational behavior of the market’s main body is the intrinsic motive, and government intervention plays an important role in regulation and guidance.

*By c*omprehensively analyzing the research on the spatial distribution of tourist towns, it is clear that current research is limited to the spatial perspective and lacks in-depth exploration of the time dimension. Most analyses of the factors that influence the spatial distribution of tourist towns were based on simple qualitative and statistical analyses, and lacked rigorous analysis. Extant research cannot reveal the degree of influence of factors on the spatial distribution of tourist towns, nor can it reveal the mechanisms that influence the spatial distribution of tourist towns. Therefore, three specific questions were addressed in this study: (1) What are the spatiotemporal differentiation characteristics of tourist towns? (2) What is the degree of influence of each factor on spatiotemporal differentiation in tourist towns? (3) What mechanisms influence the spatiotemporal differentiation of tourist towns? These questions have not been discussed in depth previously. The geographic detector is a statistical method used to explore stratified spatial differentiation and reveal the influence of its associated factors, which can reveal the mechanisms that influence the spatiotemporal differentiation of tourist towns in a scientific manner [37]. Therefore, taking Fujian, China as a typical case, this study introduces a geographic detector to explore the mechanisms that influence the spatiotemporal differentiation of tourist towns.

## 3. Materials and methods

### 3.1 Study area

Fujian Province, located on the southeast coast of China, is the starting point of the Maritime Silk Road, as well as a maritime trade center. It has a land area of 124,000 square kilometers and a sea area of 136,000 square kilometers, with nine prefecture-level cities, 11 county-level cities, 42 counties, and 31 municipal districts. The terrain of Fujian Province is high in the northwest and low in the southeast. Mountains and hills occupy approximately 90% of this area. The region spans the four major water systems of the Min, Jin, Jiulong, and Ting Rivers, and has a subtropical maritime monsoon climate. By the end of 2021, Fujian had a permanent population of 41.87 million, a GDP of 4.88 trillion yuan, a total general public budget revenue of 574.384 billion yuan, and a per capita disposable income of 51,140 yuan for urban residents and 23,229 yuan for rural residents.

Relying on rich natural ecological and cultural resources, Fujian Province has accelerated the high-quality development of the tourism economy, and the brand "Fresh Fujian" has been fully launched. By the end of 2021, Fujian had received 651,100 inbound tourists throughout the year, and the foreign exchange income from international tourism amounted to 492 million U.S. dollars; the number of domestic tourists reached 406.8051 million, the domestic tourism revenue reached 486,234 million yuan, and the total tourism revenue was 489,404 million yuan. Fujian attaches significant importance to the construction of tourist towns. In terms of policies, Fujian Province has successively issued the Three-Year Action Plan for Rural Tourism, "Hundreds of Towns and Thousands of Villages" (2014–2016), and the Three-Year Action Plan for the Quality Upgrading of Rural Tourism, "Hundreds of Towns and Thousands of Villages" (2018–2020), actively promoting the upgrading of tourist towns. As a result, the number of tourist towns in Fujian Province rapidly increased from eight in the initial construction stage in 2011 to 62 in 2014 and 140 in 2017. Subsequently, it entered the quality-improvement stage, increasing to 155 by 2020. There has been no addition to the number after 2020.

Fujian Province was chosen as an example for the following reasons: (1) Fujian Province is the first National Ecological Civilization Pilot Zone in China, and its superior ecological environment and rich regional culture provide unique conditions for the development of tourist towns. (2) Tourist towns in Fujian Province have been in the preliminary construction stage since 2011. During the 12 years of construction, there were clear temporal clues and spatial veins in the development and evolution of tourist towns. (3) There are four types of tourist towns in Fujian Province: resource-oriented, tourism reception, characteristic industrial, and ecological human settlements [11]. These types represent the typical characteristics of tourist towns’ value chain elements of tourist towns. These areas are representative of tourist towns in similar regions. Research on the influence of spatiotemporal differentiation has important reference significance and theoretical application value in the planning and construction of tourist towns.

### 3.2 Variable selection

The spatial distribution of tourist towns in Fujian Province is the result of a combination of many factors. Based on previous research and the reality of the spatial distribution of tourist towns in Fujian Province, the following seven indicators were selected as explanatory variables influencing the spatial distribution of tourist towns in Fujian Province (Table 1):

**Table 1:** The explanatory variables.

Number	Indicator	Explanation
1	Policy guidance	It enables tourism products to gather in advantageous regions and has a strong guiding effect on the spatial form of small towns [38–40]. It is an important guarantee for the industrial development of small towns, represented by per capita public budget expenditure [41].
2	Socioeconomic basis	It is an important driving force for the development of tourist towns. It can provide certain financial and technical support for the construction of tourist towns and affect the geographical spatial distribution and evolution of tourist towns [42,43]. It is represented by per capita GDP [44].
3	Industrial structure	It can play a role in the continuous optimization and coordination of the spatial organizational model in the town [30,45]. It is represented by the proportion of tertiary industry in GDP.
4	Population distribution	It is a key factor affecting the spatial differentiation of tourist towns, providing sufficient customers and human capital for the construction of tourist towns [46]. It can be represented by population density.
5	Level of urbanization	The construction of urbanization provides infrastructure, social services, industrial coordination, and other supports for the construction of tourist towns [21]. It can be represented by the urbanization rate [47].
6	Tourism resource endowment	It is the basis for the development of tourist towns. A-level scenic spots, which were approved by the Department of Culture and Tourism, have good resource foundations and supporting facilities. They are conducive to the construction of tourist towns [44,48]. This variable can be represented by the number of A-level scenic spots [49].
7	Traffic network	It is not only an important channel connecting the towns but also an important factor affecting the spatial structure [50,51]. It is represented by highway mileage [52].

### 3.3 Data source and processing

**Fig 1:**
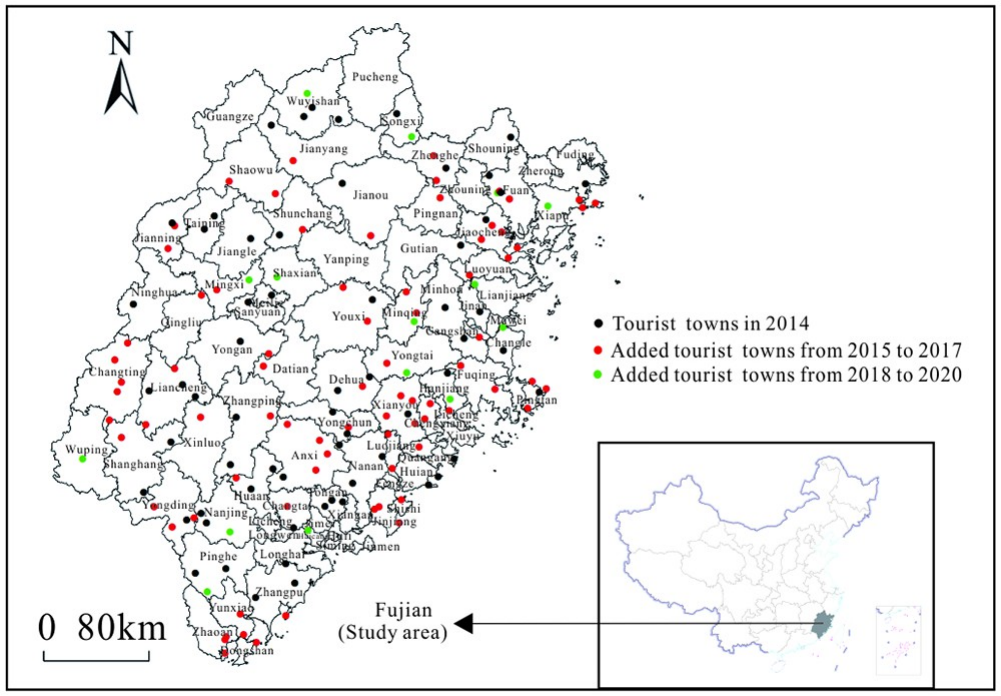
Spatial distribution of tourist towns in the study area. Contains data from standard map service of China (http://bzdt.ch.mnr.gov.cn/).The approval number of the map of China is GS(2023)2767, and the approval number of the map of Fujian is GS(2019)3333. They are freely available.

The list of tourist towns in Fujian Province was based on data from national and provincial tourist towns with characteristic landscapes, provincial demonstration towns for leisure agriculture, national and provincial tourism characteristic towns, and provincial rural tourism and leisure market towns announced by official government departments by the end of December 2021. Overall, there were 155 samples. The administrative boundary data of the samples were obtained from the National Basic Geographic Information System (NBGIS) database(http://www.geodata.cn). The geographic coordinates of the tourist towns in Fujian Province were determined using Google Earth, and a spatial distribution map of tourist towns in Fujian Province was obtained using ArcGIS visualization (Fig 1). These statistics do not include Kinmen County because of a lack of relevant data.

The years 2014, 2017, and 2020 were selected as the key time nodes of the initial development stage, rapid development stage, and quality enhancement stage of tourist towns in Fujian Province, to enable longitudinal comparison.

For the independent variables, data on A-level scenic spots in different cities were collected from the official website of the Fujian Provincial Department of Culture and Tourism. As A-level scenic spots of different levels have different radiation effects, the numbers of 5A, 4A, 3A, and 2A scenic spots were assigned weights in the ratio of 5:4:3:2 to obtain the number of A-level scenic spots, according to the practices of other researchers [52]. The remaining variables were selected from the "China Urban Statistical Yearbook," "Fujian Statistical Yearbook," and the statistical bulletin of the national economic and social development of various counties in Fujian Province. Missing data that could not be obtained were completed using linear interpolation.

### 3.4 Research methods

#### 3.4.1. Geographical concentration index

The geographical concentration index [53] was used to analyze the degree of concentration of the spatiotemporal distribution of tourist towns in Fujian Province and reflect the spatiotemporal aggregation characteristics. The formula used is as follows:

$$G=\text{100}\times \sqrt{\sum_{i=1}^{n}{\left(\frac{x_{i}}{T}\right)}^{2}}$$ (1)

where *G* is the geographical concentration index of tourist towns in the counties of Fujian Province, *Xi* is the number of tourist towns in the ith township, *T* is the total number of tourist towns in the county. and *n* is the total number of townships. The geographic concentration index is between 0 and 100; and the higher the value, the higher the degree of concentration [41,42].

#### 3.4.2. Nearest neighbor index

The nearest neighbor index [43] was used to analyze the degree of mutual proximity between point elements of the spatial structure of tourist towns in Fujian Province and to reflect the type of spatiotemporal distribution. The formula used is as follows:

$$R=\bar{{\text{r}}_{\text{1}}}/r_{E}=\bar{{\text{r}}_{\text{1}}}/(\frac{1}{2\sqrt{n/A}})$$ (2)

where *R* is the nearest neighbor index, *n* is the number of tourist towns, $\bar{{\text{r}}_{\text{1}}}$ is the average distance between adjacent points, *r*_*E*_ is the theoretical average nearest neighbor distance when point elements are randomly distributed, and *A* is the area of the study area. When *R* = 1, the point elements are randomly distributed. When *R* > 1, the point elements tend to be uniformly distributed. When *R* < 1, the point elements tend to be distributed in a clustered state [44].

#### 3.4.3. Local correlation index

The local correlation index Getis-Ord Gi * [54] was used to analyze the spatiotemporal distribution of cold spots and hot spots in tourist towns in Fujian Province and to reflect the spatiotemporal linkage characteristics.

The formula used is as follows:

$$Gi^{*}=\frac{\sum_{j=1}^{n}W_{ij}x_{j}-\bar{x}\sum_{j=1}^{n}W_{ij}}{S\sqrt{\frac{n\sum_{j=1}^{n}{W_{ij}}^{2}-{\left(\sum_{j=1}^{n}W_{ij}\right)}^{2}}{n-1}}}$$ (3)

where *n* is the number of tourist towns in Fujian Province, *w*_*ij*_ is the spatial weighting matrix, *x*_*j*_ is the number of tourist towns in region j, $\bar{x}$ is the mean of the study area, and *S* is the standard deviation of the sample. When the *Z* value of Gi* is greater than 0, the higher the *Z* value, the tighter the hotspot clustering. When the *Z* value of Gi* is less than 0, the lower the *Z* value, the tighter the cold-spot clustering [33].

#### 3.4.4. Geographic detector

Wang, et al[45] proposed a geographic detector at the Institute of Geographic Sciences and Resources of the Chinese Academy of Sciences. It is a statistical method used to reveal the explanatory ability of various factors on spatial differentiation and the interactions among the influencing factors [47,48]. Compared to traditional statistical methods, this method has fewer constraints on assumptions and overcomes the limitations of variable processing. It has been widely used in studies related to the evolution of geographic features and spatial differentiation [49,51].

In this study, the factor detector of the geographic detector model was used to explore the mechanisms that influence the spatial differentiation of tourist towns in Fujian Province. The formula is as follows:

$$q=1-\frac{\sum_{h=1}^{L}N_{h}{\sigma_{h}}^{2}}{N\sigma^{2}}$$ (4)

where *q* is the explanatory power of influence factors on the spatial distribution of tourist towns, *N* and σ_2_ are the sample size and sample variance of the entire research area, respectively, and *N*_h_ and $\sigma_{\text{h}}^{2}$ are the sample size and sample variance of sub-level regions, respectively. The range of *q* was [0,1]. The larger the *q* value, the stronger the explanatory power of the influencing factors on the spatial distribution of tourist towns in Fujian Province.

In addition, this study also used the interaction detector of the geographic detector model to analyze whether the influences were independent and to study how the explanatory power of the spatial distribution of tourist towns in Fujian Province when each of the two influencing factors interact together [52]. The types of interactions between the independent and dependent variables are presented (Table 2).

**Table 2:** Interaction types between independent variables and dependent variables.

Basis of judgment	Interaction
q(X_1_∩X_2_) <Min(q(X_1_), q(X_2_))	Nonlinearity reduction
Min(q(X_1_), q(X_2_)) <q(X_1_∩X_2_) <Max(q(X_1_), q(X_2_))	Single-factor nonlinearity decreases
Max(q(X_1_), q(X_2_)) <q(X_1_∩X_2_)	Two-factor enhancement
q(X_1_∩X_2_) = q(X_1_) + q(X_2_)	Independence
q(X_1_) + q(X_2_) <q(X_1_∩X_2_)	Nonlinear enhancement

## 4. Results

### 4.1. Spatiotemporal differentiation of tourist towns in Fujian Province

#### 4.1.1. Spatiotemporal clustering characteristics of tourist towns in Fujian Province

**Fig 2:**
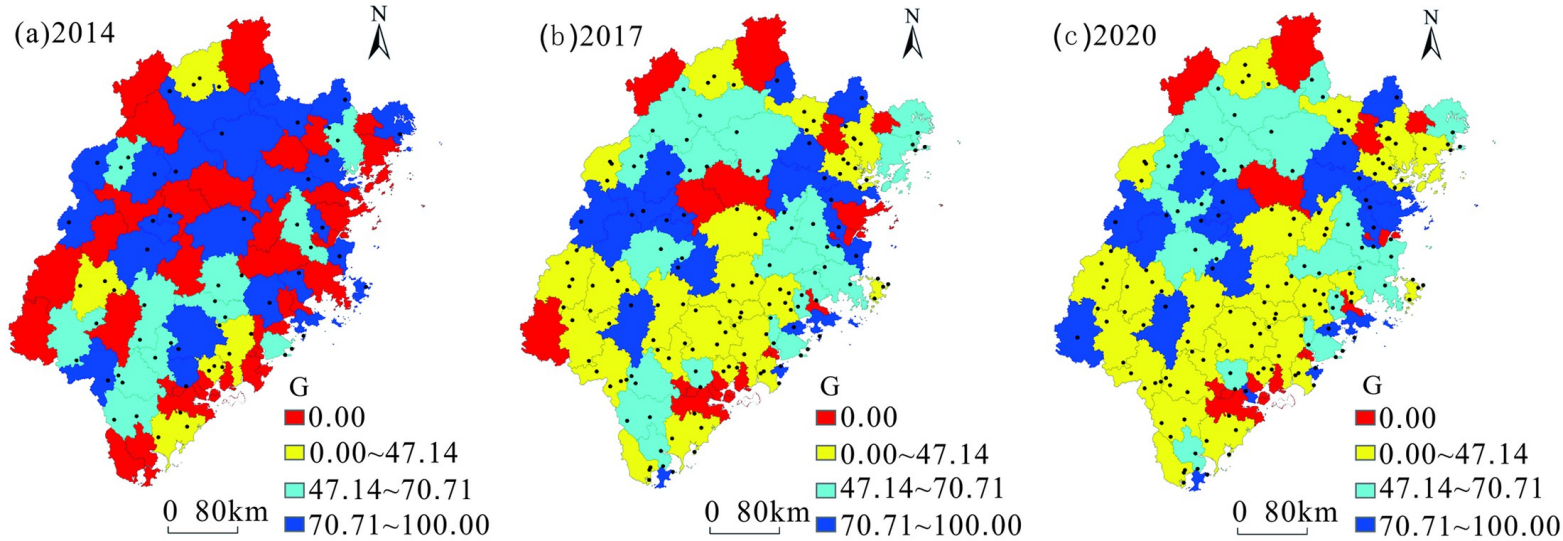
Distribution of geographical concentration index of tourist towns in Fujian Province from 2014 to 2020. Contains data from standard map service of China (http://bzdt.ch.mnr.gov.cn/). The approval number of the map of Fujian is GS(2019)3333. It is freely available.

Using ArcGIS software, the geographical concentration index G of tourist towns in Fujian Province from 2014 to 2020 was graded by natural breakpoints and was divided into four grades: low-value area (0.00), relatively low-value area (0.00–47.14), medium-value area (47.14–70.71), and high-value area (70.71–100.00; Fig 2).

Overall, the average value of the geographical concentration index of tourist towns in Fujian Province from 2014 to 2020 increased from 41.83 to 53.34, and the spatial distribution gradually became centralized. Specifically speaking, the geographical concentration index of tourist towns in Fujian Province increased significantly between 2014 and 2017. The proportion of counties in the low-value areas decreased significantly (Table 3), and they were relatively evenly distributed in various cities in Fujian Province. In contrast, relatively low-value areas increased significantly in proportion and were mainly distributed in the southern part of Fujian Province. Simultaneously, the medium-value areas increased significantly in proportion, and the main distribution areas shifted from the southern part of Fujian Province to the northern part. High-value areas shifted from the northern part of Fujian Province to parts of the counties in Fuzhou, Sanming, Ningde, and Longyan, with slightly decreasing proportion.

**Table 3:** The proportion of grade distribution and mean change of geographical concentration index of tourist towns in Fujian Province.

Year	Low-value area	Relativelylow-value area	Medium-value area	High-value area	Mean
2014	51.19%	5.95%	13.10%	29.76%	41.83
2017	26.19%	26.19%	23.81%	23.81%	51.25
2020	21.43%	32.14%	21.43%	25.00%	53.34

From 2017 to 2020, the growth rate of the geographical concentration index for tourist towns in Fujian Province decreased. Low-value areas decreased slightly in proportion, and the main distribution areas shifted from a relatively uniform distribution to counties in Nanping, Ningde, Zhangzhou, and Xiamen. The medium-value areas also decreased slightly in proportion but were still mainly distributed in the northern region of Fujian Province. The proportion of counties increased slightly in the relatively low-value areas, but they were still mainly distributed in the southern region of Fujian Province. In high-value areas, the proportion of counties increased slightly and were still mainly distributed in Fuzhou, Sanming, Ningde, and Longyan.

#### 4.1.2. Spatiotemporal distribution types of tourist towns in Fujian Province

**Fig 3:**
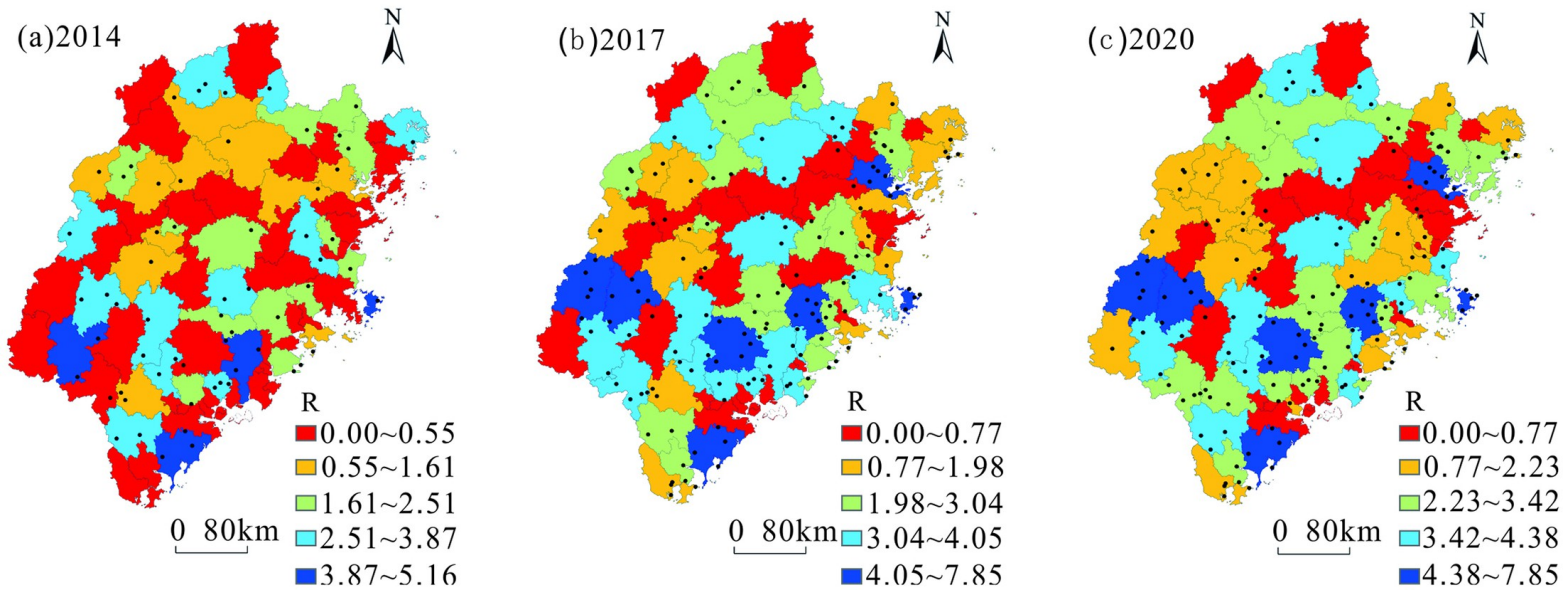
Distribution of nearest neighbor index of tourist towns in Fujian Province from 2014 to 2020. Contains data from standard map service of China (http://bzdt.ch.mnr.gov.cn/). The approval number of the map of Fujian is GS(2019)3333. It is freely available.

Using ArcGIS software, the nearest neighbor index of tourist towns in Fujian Province from 2014 to 2020 was graded by natural breakpoints and divided into five grades: low-value areas (marked in red), relatively low-value areas (marked in yellow), medium-value areas (marked in green), relatively high-value areas (marked in blue), and high-value areas (marked in dark blue; Fig 3).

In general, the average value of the nearest proximity index for tourist towns in Fujian Province from 2014 to 2020 increased from 1.14 to 2.24. The degree of proximity was significantly enhanced. The growth rate of the nearest-neighbor index was relatively high from 2014 to 2017. In the low-value areas, the proportion of counties decreased significantly (Table 4) and these were relatively evenly distributed in various cities in Fujian Province. The proportion of counties increased slightly in the relatively low-value, medium-value, and relatively high-value areas. Changes in the main distribution areas were not significant. The high-value areas increased significantly in proportion, and the main distribution areas shifted from the southern part of Fujian Province to the northern part.

**Table 4:** The proportion of grade distribution and mean change of nearest neighbor index of tourist towns in Fujian Province.

Year	Low-value area	Relatively low-value area	Medium-value area	Relatively High-value area	High-value area	Mean
2014	53.57%	13.10%	15.48%	13.10%	4.76%	1.14
2017	34.52%	17.86%	22.62%	16.67%	8.33%	2.04
2020	30.95%	25.00%	21.43%	14.29%	8.33%	2.24

The growth rate of the nearest neighbor index of tourist towns in Fujian Province slowed from 2017 to 2020. The proportion of counties decreased slightly in the low-value, medium-value, and relatively high-value areas. Changes in the main distribution areas were not significant. In relatively low-value areas, the proportion of counties increased slightly, and the main distribution areas shifted from various counties in Ningde, Fuzhou, Putian, Zhangzhou, and Sanming to departmental counties in Sanming and Fuzhou. The high-value areas remained unchanged in proportion, and the main distribution areas shifted from the northern part of Fujian Province to the southern part.

#### 4.1.3. Spatiotemporal correlation characteristics of tourist towns in Fujian Province

**Fig 4:**
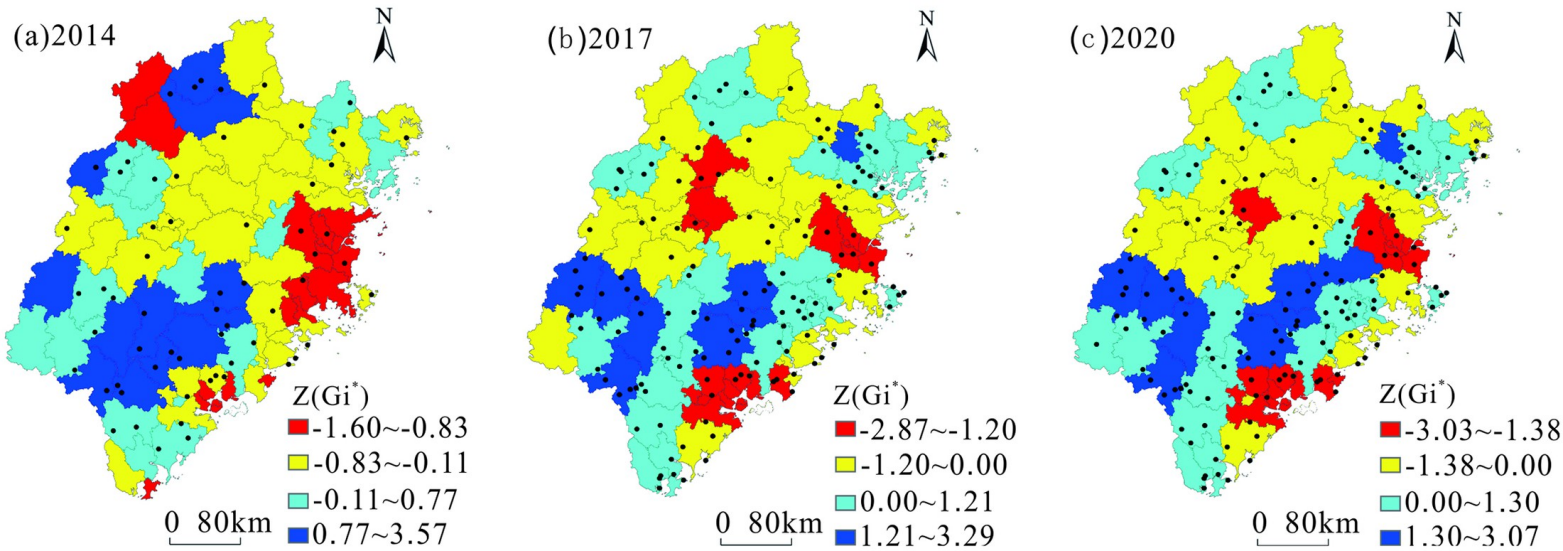
Distribution of Z-value of the local correlation index of tourist towns in Fujian Province from 2014 to 2020. Contains data from standard map service of China (http://bzdt.ch.mnr.gov.cn/). The approval number of the map of Fujian is GS(2019)3333. It is freely available.

Using ArcGIS software, the *Z*-value of the local correlation index of tourist towns in Fujian Province from 2014 to 2020 was graded by natural breakpoints, and was divided into four grades: cold spot areas (marked in red), sub-cold spot areas (marked in yellow), sub-hot spot areas (marked in blue), hot spot areas (marked in dark blue; Fig 4).

The average Z-value of the local correlation index of tourist towns in Fujian Province from 2014 to 2020 decreased from -0.13 to -0.23. This indicates that the clustering of cold spots in tourist towns in Fujian Province has become increasingly closer. The Z-value of the local correlation index decreased significantly from 2014 to 2017. The proportion of cold spot areas remained unchanged (Table 5), and the main distribution areas expanded from Fuzhou, Putian, and Nanping to Fuzhou, Xiamen, Zhangquan, Sanming, and Nanping. In the sub-cold spot areas, the proportion of counties decreased slightly, and they were mostly concentrated in the northern part of Fujian Province. In the sub-hot spots, the proportion of counties increased slightly and coverage gradually increased. The hot spot counties were primarily distributed in Quanzhou, Longyan, and Nanping, with their proportions decreasing slightly.

**Table 5:** The proportion of grade distribution and mean change of *Z*-value of the local correlation index of tourist towns in Fujian Province.

Year	Cold spot area	Sub-cold spot area	Sub-hot spot area	Hot spot area	Mean
2014	25.00%	39.29%	21.43%	14.29%	-0.13
2017	25.00%	34.52%	30.95%	9.52%	-0.23
2020	22.62%	35.71%	30.95%	10.71%	-0.23

The Z-value of the local correlation index remained constant from 2017 to 2020. The proportion of counties decreased slightly in the cold spot areas, increased slightly in the sub-cold spot and hot spot areas, and remained unchanged in the sub-hot spot areas. There was little change in the distribution of these regions.

### 4.2. Detection of factors that influence spatiotemporal differentiation in tourist towns in Fujian Province

#### 4.2.1. Detection results for q-value

Jenks’s natural discontinuity grading method was used to discretize the selected independent variables, and the geographic detector model was used for factor detection to analyze the explanatory power of the detection factors on the dependent variables. To facilitate a comparative analysis of the changes in the explanatory power of the various detection factors on the spatiotemporal differentiation patterns of tourist towns in Fujian Province in 2014, 2017, and 2020, the q-values of each detection factor were ranked (Table 6).

**Table 6:** The q-value of each detection factor.

Influencing factors	Geographical concentration index			Nearest neighbor index			local correlation index		
	2014	2017	2020	2014	2017	2020	2014	2017	2020
Policy guidance(X1)	0.063	0.014	0.055	0.030	0.140**	0.068	0.024	0.062	0.073
Socioeconomic basis(X2)	0.082	0.027	0.047	0.111*	0.104*	0.061	0.097	0.196**	0.203**
Industrial structure(X3)	0.065	0.103*	0.104	0.048	0.087	0.095	0.187**	0.191**	0.124
Population distribution(X4)	0.103*	0.105	0.103	0.072	0.091	0.108*	0.132	0.291**	0.359**
Level of urbanization(X5)	0.053	0.116*	0.039	0.069	0.139**	0.181**	0.103	0.294**	0.298**
Tourism resources endowment(X6)	0.014	0.044	0.022	0.082	0.112	0.141	0.05	0.043	0.069
Traffic network(X7)	0.127	0.158**	0.118*	0.189**	0.269**	0.271**	0.315**	0.374**	0.354**

Note:

* indicates that the significance p-value of the influencing factor is less than 0.10, which has a significant impact.

** indicates that the significance p-value of the influencing factor is less than 0.05, which has a very significant impact.

The p-value of the influencing factor without any * is greater than 0.10, which has no significant influence.

In terms of factors that influence the geographical concentration index of tourist towns in Fujian Province, the main factor in 2014 was population distribution; the main factors in 2017 were traffic network, level of urbanization, and industrial structure in order of q-value; and the main factor in 2020 was the traffic network.

In terms of factors that influence the nearest neighbor index of tourist towns in Fujian Province, the main factors in 2014 were traffic network and socioeconomic basis; the main factors in 2017 were traffic network, policy guidance, level of urbanization, and socioeconomic basis in order of q-value; and the main factors in 2020 were traffic network, level of urbanization, and population distribution in order of q-value.

In terms of factors that influence the Z-value of the local correlation index of tourist towns in Fujian Province, the main factors in 2014 were traffic network and industrial structure; the main factors in 2017 were traffic network, level of urbanization, population distribution, socioeconomic basis, and industrial structure in order of q-value; and the main factors in 2020 were population distribution, traffic network, level of urbanization and socioeconomic basis in order of q-value.

#### 4.2.2. Interaction among influencing factors

Interactive detection was executed of the factors that played a significant role in the geographical concentration index, nearest neighbor index, and *Z*-value of the local correlation index of tourist towns in Fujian Province. The results showed that the interactions between these independent variables had an enhanced relationship (Table 7). This indicates that an interaction between any two independent variables enhanced the explanatory power of the dependent variable. The spatial differentiation of tourist towns in Fujian Province is a result of the combined effects of multiple influencing factors. In terms of specific spatial distribution indicators, the geographical concentration index was primarily influenced by the level of urbanization and transportation networks; the nearest neighbor index was primarily influenced by policy guidance and traffic networks, and the Z-value of the local correlation index was primarily influenced by the socioeconomic basis and traffic network. On the time axis, the industrial structure and traffic network exhibited the strongest interaction effect in 2014, whereas the socioeconomic basis and traffic network exhibited the strongest interaction effect in 2017 and 2020. The strong interaction between traffic network and other factors has an important influence on the spatial distribution of tourist towns in Fujian Province.

**Table 7:** Interaction detection results of influencing factors on the spatial distribution of tourist towns in Fujian Province.

Geographical concentration index			Nearest neighbor index			local correlation index		
Interaction factor	Impact coefficient	Year	Interaction factor	Impact coefficient	Year	Interaction factor	Impact coefficient	Year
X5∩X7	0.395	2017	X2∩X7	0.383	2014	X3∩X7	0.415	2014
X3∩X7	0.340	2017	X1∩X7	0.393	2017	X2∩X7	0.559	2017
X3∩X5	0.271	2017	X2∩X7	0.361	2017	X3∩X7	0.516	2017
			X1∩X5	0.349	2017	X2∩X5	0.515	2017
			X5∩X7	0.347	2017	X5∩X7	0.512	2017
			X1∩X2	0.316	2017	X4∩X7	0.458	2017
			X2∩X5	0.301	2017	X3∩X5	0.455	2017
			X5∩X7	0.361	2020	X2∩X3	0.425	2017
			X4∩X7	0.305	2020	X4∩X5	0.398	2017
			X4∩X5	0.213	2020	X3∩X4	0.397	2017
						X2∩X4	0.393	2017
						X2∩X7	0.583	2020
						X2∩X4	0.511	2020
						X5∩X7	0.500	2020
						X4∩X7	0.481	2020
						X2∩X5	0.470	2020
						X4∩X5	0.414	2020

### 4.3. Mechanism influencing spatiotemporal differentiation of tourist towns in Fujian Province

Among the factors affecting the spatiotemporal aggregation characteristics of tourist towns in Fujian Province, the main factor changed from population distribution to traffic network, because in the pre-construction period of tourist towns, there is a high demand for good human resource security. Counties with higher population densities can provide sufficient human resources for the construction of tourist towns; thus, counties with higher population densities are more likely to cluster tourist towns. In the middle and late stages, the construction of tourist towns requires more effort to procure and deliver supplies and equipment and needs to better attract tourists. Counties with better transportation accessibility are clearly superior in these respects such that a greater number of tourist towns are clustered.

Among the factors affecting the spatiotemporal distribution of tourist towns in Fujian Province, the main factors changed from traffic network and socioeconomic basis to traffic network, level of urbanization and population distribution. This is because during the pre-construction period of tourist towns, counties with richer traffic networks and more developed socioeconomics can provide good financial and logistic protection for the construction of tourist towns and drive the formation of tourist towns, which tend to be more evenly distributed spatially. At a later stage, counties with richer traffic networks, higher rates of urbanization, and greater population densities can provide a good source of customers for tourist towns. Therefore, these counties are more likely to form tourist towns that are spatially evenly distributed.

Among the factors affecting the spatiotemporal correlation characteristics of tourist towns in Fujian Province, the main factors changed from traffic network and industrial structure to population distribution, traffic network, level of urbanization, and socioeconomic basis. This is because in the pre-development period of tourist towns, counties with richer traffic networks and a larger proportion of tertiary industries in GDP have closer connectivity among their townships, which can provide support for the construction of tourist towns in terms of industrial elements such as food, accommodation, transportation, tourism, shopping, and recreation, such that hot spots of distribution of tourist towns are formed. At a later stage, counties with greater population density, richer traffic networks, higher rates of urbanization, and more developed socioeconomics can deliver stable sources of customers to tourist towns, forming hot spots for the distribution of tourist towns.

**Fig 5:**
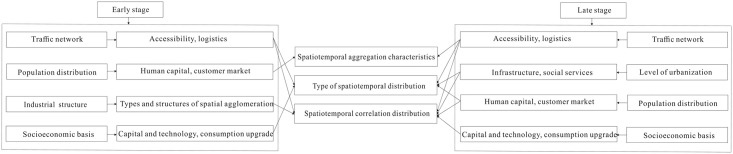
Mechanism influencing spatiotemporal differentiation of tourist towns in Fujian Province.

Based on the above analysis, mechanism influencing spatiotemporal differentiation of tourist towns in Fujian Province is shown in Fig 5.

## 5. Discussion

This study analyzed the spatiotemporal evolution of tourist towns in Fujian Province, adding the time dimension, and using the geographic concentration index, nearest neighbor index, and local correlation index with a geodetector model. The model explored the degree of influence of each factor on spatiotemporal differentiation and analyzed the mechanism influencing spatiotemporal differentiation. This study can provide research support for the construction of a spatial pattern of a reasonable tourist town and provide reference cases, which represents innovation in terms of the content and methodology of the research. This study verified the applicability of the geodetector model. The method not only enables analysis of the ability of each factor to explain spatial divergence but analysis of the interactions among the factors, which denotes a highly practical statistical analysis method.

It was found that there were not only clear spatial distribution differences but also clear temporal distribution differences among tourist towns in Fujian Province. Six factors—traffic network, level of urbanization, population distribution, industrial structure, socioeconomic basis, and policy guidance—influenced the spatiotemporal distribution of tourist towns in Fujian Province.

The traffic network, level of urbanization and population distribution were the core factors influencing the spatiotemporal differentiation of tourist towns in Fujian Province. The traffic network had the highest explanatory power and the most significant influence on the spatial differentiation of tourist towns. This result is consistent with the results of previous studies [32,34,35]. The construction of a traffic network directly determines a region’s accessibility. Transportation is an essential prerequisite for tourism development [27]. Counties with more developed traffic networks have greater accessibility and more developed logistics, which is more conducive to the delivery of materials and products. Thus, the distribution of tourist towns is also more concentrated. The level of urbanization had a dominant influence on the spatial differentiation of tourist towns. This confirms the results of previous research [30]. In counties with higher urbanization levels, the infrastructure of townships is relatively perfect, providing more employment opportunities for local farmers, and the social service function of towns is guaranteed to a certain extent, which has a radiating and driving effect on the construction and development of tourist towns [55]. Therefore, the level of urbanization has a more significant impact on the spatial layout of tourist towns. Population distribution has also been shown to be a key factor that influences the spatial differentiation of tourist towns [27,30]. Counties with higher population densities can provide sufficient human resources and passenger flow for the construction of tourist towns and create social and economic value [32]. At the same time, population density can also reduce the social consumption level, promote infrastructure construction of the town, improve the social security function of the town, and promote the economic development. Therefore, the population distribution affects the spatial distribution of tourist towns.

The industrial structure and socioeconomic basis are important factors that influence the spatiotemporal differentiation of tourist towns in Fujian Province, confirming the results of previous studies [26,33,34,45,54]. First, the construction of tourist towns involves primary, secondary, and tertiary industries; it is difficult to sustain the construction of tourist towns without these industries. Changes in the industrial structure can impact the development of urban agglomerations, which influences the type and structure of spatial agglomeration in tourist towns. Second, counties with a higher level of socioeconomic development have advantages in financing, technological bases, market operating environments, and consumption levels, which are more likely to contribute to the agglomeration of production factors in tourist towns, thus forming a scale effect of spatial agglomeration [56].

Policy guidance fundamentally influenced the spatiotemporal differentiation of tourist towns in Fujian Province. Policy guidance affected the spatial differentiation of tourist towns, confirming findings of other studies [26,31,33]. Counties with larger government public budget expenditures have advantages in infrastructure construction, public service support, and industrial policy support, which can prompt tourism products to cluster in advantageous areas and guide the development of spatial patterns of tourist towns [38–40].

In addition, this study found that tourism resource endowment had no significant impact on the spatial distribution of tourist towns in Fujian Province. This result is inconsistent with the results of previous studies [33,34]. This is because tourist towns in Fujian Province are resource oriented, ecological-settlement oriented, tourism-reception oriented, and characteristically industry oriented. Fujian does not rely solely on tourism resources for the development of tourist towns. Therefore, the impact of tourist resource endowment on the spatial distribution of tourist towns in Fujian Province was not significant.

This study has some limitations. First, this study explored the spatiotemporal distribution of tourist towns based on a provincial scale using the geographical concentration index, nearest neighbor index, and local correlation index, which may not fully reflect the spatial distribution characteristics of tourist towns. Because the spatial distribution characteristics of tourist towns are multidimensional, in the future, the distribution characteristics of tourist towns should be measured more comprehensively based on national, municipal, and tourist circle scales, combined with other dimensional indicators, such as the coefficient of variation and kernel density estimation. Second, the selection of the factors and indicators for the spatiotemporal differentiation of tourist towns may not have been comprehensive. In addition to the factors discussed in this study, the impact of geographical conditions, culture, and other factors also needs to be further explored in future work.

## 6. Conclusion and recommendations

### 6.1 Conclusion

This study explored the spatiotemporal evolution of tourist towns in Fujian Province using the geographical concentration index, nearest neighbor index, and local correlation index, and revealed mechanisms of influence using a geographic detector. The results revealed that the spatial distribution of tourist towns in Fujian Province tends to concentrate over time, the degree of proximity is enhanced, and the clustering of cold spots becomes closer. Among the factors affecting the spatiotemporal aggregation characteristics of Fujian Province tourist towns, the main factor in the early stage was population distribution, whereas the main factor in the later stage was the traffic network. Among the factors influencing the spatiotemporal distribution of tourist towns in Fujian Province, the main factors in the early stage were the traffic network and socioeconomic basis, while the main factors in the late stage were the traffic network, level of urbanization, and population distribution. Among the factors affecting the spatiotemporal correlation characteristics of tourist towns in Fujian Province, the main factors in the early stage were the traffic network and industrial structure, whereas the main factors in the later stage were population distribution, traffic network, level of urbanization, and socioeconomic basis. Among the factors that affected the spatiotemporal differentiation of tourist towns in Fujian Province, traffic network, level of urbanization, and population distribution were the core factors, while industrial structure and socioeconomic basis were important factors, and policy guidance was the fundamental factor.

These six factors interacted with each other and jointly affected the spatiotemporal differentiation of tourist towns in Fujian Province. In the construction of tourist towns, decision-makers should continuously optimize the overall transportation pattern, improve the supporting facilities of towns, integrate the elements of the tourism industry, and improve the construction of the institutional environment. This study provides a theoretical reference for the future development of tourist towns.

### 6.2 Recommendations

Based on the above analysis, to promote the optimization of the spatial layout of tourist towns in Fujian Province and other similar areas around the world, the government should first focus on the construction of a comprehensive transportation network to form a "large transportation" pattern and enhance the accessibility of tourist towns. Second, it is important to promote the construction of new-type urbanization; coordinate the arrangement of town infrastructure, municipal facilities, safety and security and other public services; improve industrial support and social service functions; enhance the level of urbanization; and promote the increase of population density. This would provide a foundation of human resources and sources for the development of tourist towns, and radiate and drive the construction of such towns. Third, the industrial structure should be optimized to strengthen the comprehensive integration of agriculture, industry, culture, tourism, recreation, sports, and other related fields and to promote the industrial spatial agglomeration of tourist towns. Fourth, investments should be sought from all sectors of society, actively cultivating business forms, stimulating social consumption, consolidating the socioeconomic basis, and contributing to the agglomeration of production factors and industrial spaces in tourist towns. Fifth, China should strengthen the guidance of industrial policies, increase public budgetary expenditures for the construction of tourist towns, advance the market-oriented allocation of tourism resources, and promote the integration and synergistic development of regional resources.

## Supporting information

S1 File (XLSX)

S2 File (DOCX)
